# A multi-step completion process model of cell plasticity

**DOI:** 10.1093/bib/bbaf165

**Published:** 2025-04-14

**Authors:** Chen M Chen, Rosemary Yu

**Affiliations:** Department of Molecular Developmental Biology, Radboud Institute for Molecular Life Sciences, Faculty of Science, Radboud University, Geert Grooteplein-Zuid 26-28, Nijmegen, 6525GA, The Netherlands; Department of Molecular Developmental Biology, Radboud Institute for Molecular Life Sciences, Faculty of Science, Radboud University, Geert Grooteplein-Zuid 26-28, Nijmegen, 6525GA, The Netherlands

**Keywords:** cell plasticity, completion process, mathematical modeling, time-series omics data

## Abstract

Plasticity is the potential for cells or cell populations to change their phenotypes and behaviors in response to internal or external cues. Plasticity is fundamental to many complex biological processes, yet to date there remains a lack of mathematical models that can elucidate and predict molecular behaviors in a plasticity program. Here, we report a new mathematical framework that models cell plasticity as a multi-step completion process, where the system moves from the initial state along a path guided by multiple intermediate attractors until the final state (i.e. a new homeostasis) is reached. Using omics time-series data as model input, we show that our method fits data well; identifies attractor states by their timing and molecular markers which are well-aligned with domain knowledge; and can make quantitative and time-resolved predictions such as the molecular outcomes of blocking a plasticity program from reaching completion, to an R^2^ of 0.53–0.63. We demonstrate that application of our model to primary patient-derived data can provide quantitative insights and predictions that may be useful in guiding further research and potential biomedical interventions.

## Introduction

Cells and cell populations are highly plastic, meaning that they can robustly respond to a variety of signals and (non-lethal) perturbations by changing their characteristics and behaviors [1, 2]. These phenotypic changes arise through a cascade of molecular events over time, and is ‘complete’ when a new homeostasis is reached [2, 3]. To study plasticity, it is common to systematically track the time course of these molecular changes by collecting omics (e.g. transcriptomics, proteomics) time-series data. These changes often exhibit complex non-linear, non-monotonous dynamics, posing a significant challenge for mathematical modeling of plasticity at the omics level. In many studies of cell plasticity where time-series omics data are collected, formal mathematical analysis of the molecular changes over time is restricted to various local/piecewise polynomial regression models, either prior to or following dimensionality reduction (e.g. refs [4–6]). These models are useful for visualizations of data patterns or for statistical testing, but their parameters are uninterpretable as meaningful biological concepts or events that may represent causal mechanisms underlying the plasticity program under study. More sophisticated methods to extract information from omics time-series data, such as time-lagged correlation [7], molecular event inference [8], and interaction/regulatory network reconstruction [9], can be used to infer causal mechanisms, but lack the ability to make predictions that are quantitative and/or time-resolved. As such, conclusions drawn from these analyses are difficult to validate and translational application remains limited.

**Figure 1:**
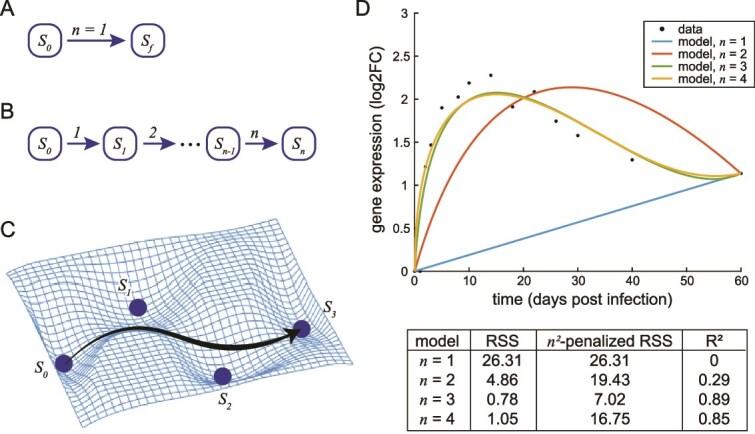
Modeling cell plasticity as multi-step completion processes. (A) Schematic of the simplest completion process, where the system reaches the final state in single step ($n=1$). (B and C) Schematic of an $n$-step completion process where the system moves along a path guided by $n-1$ intermediate attractors, until reaching the final state. (D) Best fit models of $n=1,\dots, 4$, of a single gene (*Il4i1*) from ref [16]. Data points are expression levels of *Il4i1* in the mouse lung for 60 days following a viral infection (ref [16]). Colored lines are fitted CP models for the single gene. The RSS, *n^2^*-penalized RSS, and R^2^ of each fitted model are given.

Many molecular programs related to plasticity can be viewed as a multi-step completion process: the system moves, over time, from the initial state along a path guided by multiple intermediate attractors, until the final state is reached [10–14] (Fig. 1A–C). Mathematical techniques for modeling and analyzing such processes, pioneered in physics and biophysics, have thus far been focused on modeling the time that it takes for a process to reach completion [10–14]. Examples of applications of these modeling frameworks include, in virology, asking how long it takes for a virus-infected cell to start producing new viral particles [14] and, in cellular signal transduction, asking how long it takes for cells to mount a response to an external signal [10]. In these types of problems, the identity of the intermediate attractor states are either known, assumed, or simply not of interest. However, in many areas of plasticity research, it is often of interest to identify the intermediate attractor states by their molecular markers. Potential applications of these insights include potential medical applications in disease progression or wound healing, for example in determining, for a particular patient at a particular time, which state the disease or healing process has progressed to; or whether the progression to the next state can be prevented (in the case of disease) or accelerated (in the case of healing). Intuitively, it should be possible to gain such insights through the commonly collected omics time-series data, but mathematical techniques to do so are so far lacking.

Here we describe a new mathematical model of cell plasticity as a completion process, which models the molecular changes occurring during the process (i.e. omics time-series data) as a function of the stochastic probabilities of reaching sequential attractor states over time. Importantly, we show that our developed control point (CP) model accurately identifies attractor states by their molecular markers and timing, in a fully data-driven manner. Moreover, the CP model can be used to make non-trivial predictions about the modeled plasticity program. For example, to date no modeling framework is able to predict the molecular outcomes of a plasticity process if it is interrupted and blocked from reaching completion. We show that the CP model can make such predictions in a quantitative and time-resolved manner, to an R^2^ of 0.53–0.63. We anticipate that the application of the CP model to diverse plasticity programs will provide quantitative insights and predictions that can be used in biomedical and bioengineering applications.

## Results

### Model description

Consider some plasticity program that can be represented by a completion process as shown in Fig. 1A–C, where each attractor state $S$ is ‘marked’ by a particular set of molecular characteristics. We model this process as $n$ discrete state transitions, i.e. $n$ independent Bernoulli trials, where a ‘success’ means that the system proceeds to a particular state, while a ‘fail’ means that the system stays in a previous state. At any moment during the progression of the process ($\tau \in \left[0,1\right]$), the probability of successfully undergoing exactly $k$ state transitions, with $k=0,\dots, n$, is given by the binomial distribution [15] (see Methods section, Equation 1). Suppose that omics data (e.g. transcriptomics) is collected over time, at enough timepoints to be fully representative of the completion process. Then, for each molecular characteristic $g$ measured over time (e.g. abundance of a transcript), we find a model of a size of $n$ that fits the measurement data arbitrarily well (Fig. 1D), and solve for a set of CPs ${P}_g$ that specifies this model (see Methods section, Equation 2). Then, for all measured molecules $G\ni g$, we use the collective CPs ${P}_G$ to infer the intermediate attractor states (${S}_k,k=1,\dots, n-1$) by both their timing ($t$) and their molecular markers (a subset of the measured molecular characteristics $G$). Note that at $k=0$ and $k=n$, attractors ${S}_0$ and ${S}_n$ represent the initial and final states of the process, respectively, and their markers are taken as all measured molecules $G$; see details in the Methods section. Below we illustrate these concepts, and benchmark the CP model, using three molecular programs in plasticity as examples.

### Modeling a viral infection-induced immune plasticity program

**Figure 2:**
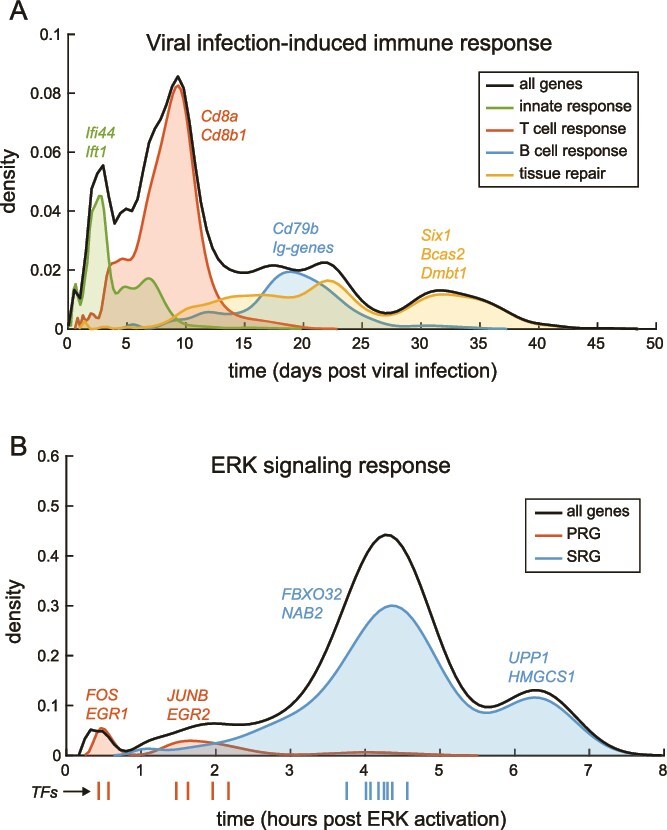
The CP model accurately recapitulates known phases of plasticity programs, including their timing and molecular markers. (A) CP model-identified attractor states accurately recapitulates known phases of a viral infection-induced response in the mouse lung (ref [16]), by their timing and molecular markers. (B) The CP model accurately recapitulates known phases of the ERK signaling-induced gene expression in HEK293 cells (ref [24]) by their timing and molecular markers, and discovers temporally and functionally distinct attractor states that are previously unresolved. TFs are subsetted and shown as a strip plot below the x-axis, marked by an arrow and color-coded by PRG or SRG as shown in the figure legend.

In the first example, we model a viral infection-induced gene expression response in the mouse lung [16], representing a plasticity program at the tissue level. Response to a viral infection is a textbook example of a multi-step completion process, consisting of four phases between the initial state (${S}_0$) and final recovery to homeostasis (${S}_n$): first the three phases of the immune response (innate response, T cell response, and B cell response), followed by a fourth phase of tissue repair [17]. To model this process, we mined a set of transcriptomics time-series data of the mouse lung collected after a non-lethal infection with influenza A over a period of 60 days [16]. We find the best-fit model for each gene $g$ over time (see Fig. 1D for an example, and see the GitHub repo for similar graphics of additional genes), and use a kernel density plot for a global overview of the positioning of the CPs ${P}_G$ of all genes along the time-axis (Fig. 2A). The CP model accurately captures that this plasticity program is best described by a completion process of 4 intermediate attractor states between the initial state ${S}_0$ and the final state ${S}_n$ (Fig. 2A), in agreement with the known four phases of the viral infection-induced response. Moreover, the model-identified molecular markers of each attractor state are fully in agreement with known markers of the different phases of the viral infection-induced response (Fig. 2A). This includes *Ifi44* and *Ift1* as markers of the first attractor state (innate response) [18, 19]; *Cd8a* and *8b1*, classic markers of cytotoxic T cells [17], marking the second attractor state; the B cell marker *Cd79b* [20] and 14 out of 16 detected *Igh*, *Igj*, and *Igk* genes indicative of antibody production during the B cell response [17], marking the third attractor state; and *Six1*, *Bcas2*, and *Dmbt1* marking the final attractor state of tissue repair [21–23]. Finally, the timing of each phase of the response, modeled in a completely data-driven manner (Fig. 2A), perfectly recapitulates schematics of the host immune response over time that can be found in textbooks [17].

### Modeling the extracellular signal-regulated kinase signaling response program

In the second example, we use the CP model to study extracellular signal-regulated kinase (ERK) signaling-induced gene expression in HEK293 cells [24]. ERK is the final kinase in the classic RAF–MEK–ERK signaling pathway. Upon activation of ERK signaling, transcriptomic changes of HEK293 cells (and other cell lines) occur through two phases: first the induction of a set of genes called primary response genes (PRGs), followed by the induction of secondary response genes (SRGs) which is dependent on the translation of the PRGs [24, 25]. To model this program, we mined a set of ERK signaling-driven transcriptomics time-series data in HEK293 cells, covering a span of 10 h [24]. The CP model identifies two attractor states marked by PRGs, followed by two attractor states marked by SRGs, recapitulating the known phases of ERK signaling (Fig. 2B). Interestingly, this analysis shows that the SRGs, which are previously thought of as a single group of genes characterized by their dependence on PRG translation, are in fact marking two temporally distinct attractor states (Fig. 2B). The earlier of the two SRG-marked attractor states, at 3.5–5 h after ERK signal induction, is marked by genes such as *FBXO32* and *NAB2*, which are negative regulators of ERK signaling and/or its downstream effectors [26–28]. The later attractor state, at 6–7 h after ERK signal induction, is marked by genes such as *UPP1* and *HMGCS1*, which are metabolic enzymes supporting cell proliferation [29, 30]. The discovery of these temporally and functionally distinct attractor states therefore suggests a division of the SRGs into two subsets for further molecular characterization—similar to what has been done in the investigation of the PRGs [24, 25].

We further note that both of the PRG-marked attractor states are comparatively narrow in terms of the global positioning of its CPs ${P}_G$ on the time-axis, while those of the SRG-marked attractor states are comparatively broad. Moreover, we find that a large fraction of genes in the PRG-marked attractor states are transcription factors (TFs; 67% for the first and 14% for the second PRG-marked states, compared to 6% and 0% in the SRG-marked states). Subsetting this dataset for TFs, we observe that CPs of TFs cluster along the time-axis in line with the model-identified attractor states (Fig. 2B, strip plot below the x-axis). Moreover, even in the SRG-marked attractor state, the TF CPs fall within a narrow band on the time-axis, comparable to TFs in the PRG-marked attractor states (Fig. 2B, strip plot below the x-axis). This is consistent with the current view of the regulation of ERK signaling response as a highly coordinated series of sequential TF activation [24, 25], again demonstrating that our CP model can identify biologically relevant attractor states from omics time-series data, in a fully data-driven manner.

### Quantitative and time-resolved predictions by the control point model

**Figure 3:**
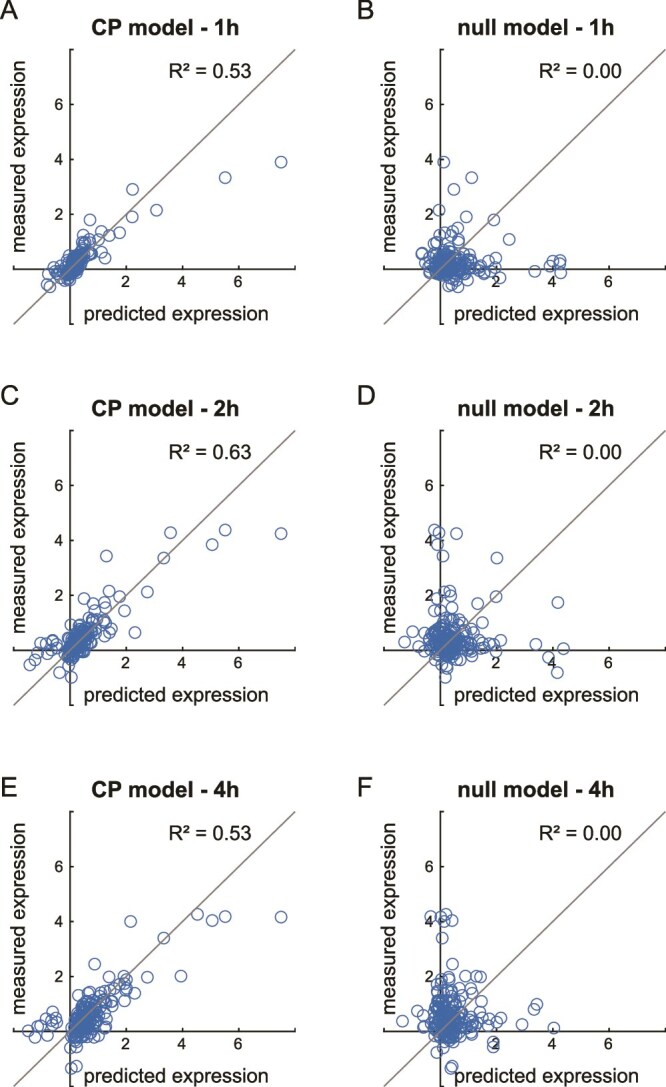
Non-trivial predictions of the CP model. (A, C, and E) Comparison between model-predicted and experimentally collected gene expression in the ERK signaling-induced gene expression program, when cells are treated with the protein translation inhibitor CYHX. The experimentally collected data are mined from ref [24]. (B, D, and F), comparison between a null model and experimental data from ref [24].

Previous methods to identify the phases of ERK signaling-induced gene expression (e.g. peak expression time [25]) are not able to make non-trivial predictions. Here we show that the CP model can quantitatively predict the molecular outcome of the modeled completion process, if the process is interrupted and blocked from reaching completion. Conceptually speaking, interrupting the completion process at step $k$ (i.e. interrupting the transition from ${S}_{k-1}$ to ${S}_k$) would predict that the system becomes attracted to the preceding state, ${S}_{k-1}$, as the new stable attractor ${S}_n^{\prime }$ (see Fig. 1B and C). Using the CP model, the dynamics of the resulting molecular program can be predicted in a time-resolved manner. To illustrate this, we used a published dataset where HEK293 cells are co-treated with an ERK activation signal and a protein synthesis inhibitor for 1, 2, and 4 h [24]. The protein synthesis inhibitor blocks the translation of PRGs into proteins, thereby preventing the induction of SRGs which marks the next attractor state(s) [24]. Using these experimental gene expression measurements as validation, we demonstrate that the CP model predictions are quantitative, time-resolved, and accurate to an R^2^ of 0.53–0.63 (Fig. 3A, C, and E), a dramatic improvement to the null model which is completely unable to make such predictions (R^2^ = 0, Fig. 3B, D, and F).

### Modeling the differentiation of primary epidermal keratinocytes

**Figure 4:**
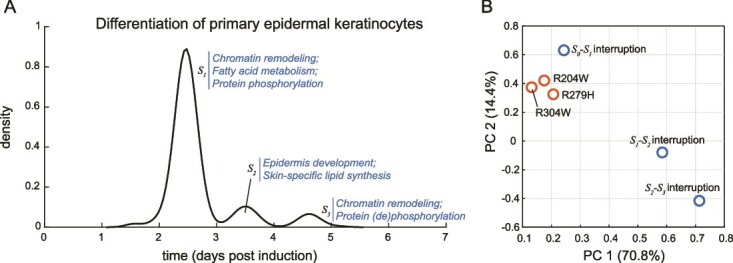
The CP model identifies previously unknown attractor states of the keratinocyte differentiation process. (A) CP model-identified attractor states of the differentiation process of keratinocytes with wild-type p63 (derived from a healthy volunteer). The experimentally collected data are mined from ref [33]. DAVID gene ontology terms (ref [34, 35]) of markers of each attractor state is shown. (B) Comparison between model-predicted gene expression profile of three hypothetical disease scenarios, and three gene expression profiles of keratinocytes derived from EEC syndrome patients carrying p63 mutations. The EEC syndrome patient-derived data are mined from ref [37].

Finally, we apply the CP model to a primary patient- (and healthy volunteer-) derived dataset of a relatively understudied completion process related to a rare disease, to demonstrate the potential of the CP model in providing quantitative insights and predictions that may be useful in guiding further research and biomedical interventions. The TF p63 is a key regulator of keratinocyte differentiation during development, the disruption of which can lead to a spectrum of developmental disorders such as the Ectrodactyly-Ectodermal dysplasia-Cleft (EEC) syndrome [31]. Previous work has shown that the differentiation of primary keratinocytes with wild-type p63 (derived from healthy volunteers), is a completion process that reaches completion in 7 days [32]. We apply the CP model to a time-series transcriptomics dataset of this ‘normal’ keratinocyte differentiation process [33]. We identify 3 intermediate attractor states in this process (Fig. 4A): ${S}_1$, occurring at d2–3 after induction of differentiation, marked by genes involved in chromatin remodeling, fatty acid metabolism, and protein phosphorylation; followed by ${S}_2$, occurring at d3–4, marked by genes specific to epidermis development and skin-specific lipid synthesis (ceramides, sphingolipids, and others); and finally ${S}_3$, occurring at d4–5, marked by genes involved in chromatin remodeling and protein (de)phosphorylation (Fig. 4A; full gene list and gene ontology [34, 35] can be found in the GitHub repo). These discoveries confirm the previously reported roles of epigenetic landscape rewiring [32] and skin-specific lipid metabolism [36] in keratinocyte differentiation, and further extends our knowledge of this process by providing a time-resolved sequential order of these events, which has not been possible before (Fig. 4A).

We then use the CP model to predict the molecular outcomes of disrupting the keratinocyte differentiation process. Specifically, we predict the transcriptomic expression at day 7 post induction, for three hypothetical disease scenarios: (i) where the normal keratinocyte differentiation process is interrupted at the ${S}_0$-${S}_1$ transition; (ii) where the process is interrupted the ${S}_1$-${S}_2$ transition; and (iii) where the process is interrupted at the ${S}_2$-${S}_3$ transition (Fig. 4B). We compare these predications of hypothetical disease scenarios to a true disease scenario: the differentiation of keratinocytes derived from EEC syndrome patients, carrying hotspot p63 mutations (R204W, R279H, or R304W) [37]. We find that the measured gene expression profile of EEC syndrome patient-derived keratinocytes, at day 7 post induction of differentiation [37], all cluster closely to the predicted disease scenario of an ${S}_0$-${S}_1$ disruption in the keratinocyte differentiation process (Fig. 4B). These results indicate that an ${S}_0$-${S}_1$ disruption in the keratinocyte differentiation process is likely causal to the development of EEC syndrome. In light of these findings, interventions targeting the restoration of ${S}_0$-${S}_1$ transition in keratinocyte differentiation should be explored in further research as potential treatment for this disease.

## Discussion

Here we describe a new modeling framework for cell plasticity, which models the molecular changes over time in a plasticity program as a multi-step completion process (Fig. 1). Using omics time-series data as input, the CP model accurately identifies the attractor states of a complex plasticity program by their timing and molecular markers, in a completely data-driven manner. We benchmark the CP model using three molecular programs in plasticity, showing that the model fits data well and identifies attractor states that are well-aligned with prior knowledge (Fig. 2). Moreover, CP model-based analysis can lead to new knowledge discovery, for example showing that SRGs in the ERK signaling-induced gene expression program can be divided into two subsets, marking two attractor states that are functionally and temporally distinct (Fig. 2). Finally, we show that application of the CP model to primary patient-derived datasets can provide quantitative insights and predictions that may be useful in guiding further research and biomedical interventions, such as the disruption of keratinocyte differentiation in EEC syndrome patients (Fig. 4).

We note that the CP model is not specifically built to analyze keratinocyte differentiation (or any other plasticity program in particular), yet it is able to cogently connect previously disparate observations of epigenetic landscape rewiring [32] and skin-specific lipid metabolism [36] in keratinocyte differentiation, using only transcriptomics data. Thus, the CP model is the first description of a generalized modeling framework for cell plasticity, which can make non-trivial predictions in a quantitative and time-resolved manner. We believe that this is the most important property of a useful model: the ability to not only accurately describe the molecular behaviors of a cell, but also to predict and eventually use these predictions in bioengineering or biomedical applications. Here, we demonstrate that the CP model can accurately predict the molecular outcomes of blocking a completion process from reaching completion, in a quantitative and time-resolved manner (Figs 3 and 4B). As cell plasticity is fundamental to a wide range of complex biological processes, we anticipate that the CP model will provide important insights in the molecular basis of these processes in the future.

## Methods

### Model description

Consider an $n$-step, single-path completion process as shown in Fig. 1A–C. We model the $n$ state transitions as $n$ independent Bernoulli trials, where a ‘success’ means that the system proceeds to a particular state, while a ‘fail’ means that the system stays in a previous state. At any moment in the progression of the process, $\tau \in \left[0,1\right]$, the probability of successfully undergoing exactly $k$ state transitions, with $k=0,\dots, n$, is given by the binomial distribution [15], ${B}_{n,k}\left(\tau \right)$:


(1) \begin{equation*} {B}_{n,k}\left(\tau \right)=\left(\genfrac{}{}{0pt}{}{n}{k}\right){\tau}^k{\left(1-\tau \right)}^{n-k}. \end{equation*}

The mathematical expression of the entire $n$-step completion process is:


(2) \begin{equation*} p\left(\tau \right)=\sum_{k=0}^n{B}_{n,k}\left(\tau \right)\cdot{P}_k, \end{equation*}

where $p\left(\tau \right)$ denotes the molecular characteristics (omics) of the system over time (to be fitted), and ${P}_k$ denotes the (fitted) $k$^th^ CP of the molecular characteristics. Note that ${B}_{n,k}\left(\tau \right)$ is a complete basis (see Bernstein’s proof of the Weierstrass approximation theorem, ref [38, 39]), thus every dataset can be fitted arbitrarily well by some (possibly very complex) CP model. Additionally, $p\left(\tau \right)$ is uniquely determined by its CPs ${P}_k$: if two functions $p\left(\tau \right)$ and ${p}^{\prime}\left(\tau \right)$ are identical, then${P}_k={P}_k^{\prime }$ by definition [40]. In practice, we fit the CP model to each molecular characteristic $g$ independently (i.e. solving for each $g$ a set of CPs $\left\{{P}_g\right\}={P}_{g,k},k=0,\dots, n$), since not all characteristics are expected to be a marker for every intermediate state. The intermediate states of the entire completion process are then inferred through collective analysis of the characteristic-specific CPs, described in the sections below.

### Input data and pre-processing

*Input data.* As input data, we take any omics time-course dataset of plasticity that satisfies the following: (1) the dataset contains the initial homeostasis as an unperturbed control sample at ${t}_0$; (2) the dataset contains the final homeostasis (which does not need to be the final timepoint, see ‘Identifying time of final expression level’ section below); and (3) the dataset contains sufficient ‘middle’ timepoints to reasonably assume that the completion process is represented in full. In principle, single-cell molecular data such as single-cell RNA sequencing, either as a time-series dataset or following pseudo-time analysis, can also be modeled by the CP model. However, here we refrain from this application as single cell data is currently known for high technical and analytical variabilities leading to unstable quantitative results [41]. In this paper, we mined transcriptomics time-series data as input data from refs [16, 24, 33, 37], as described in the Results section.

*Feature selection.* In an omics time-series dataset, we expect that a subset of molecular characteristics (e.g. a subset of transcripts in a transcriptomics dataset) will not exhibit significant changes over time. These features are not of interest in our modeling framework, as they would not be markers of any intermediate state. Feature selection is therefore performed by differential expression analysis using standard practices, comparing the expression data of each feature $g$ at all timepoints to the unperturbed control sample at ${t}_0$. The selection criteria typically includes a minimum fold-change cutoff and a minimum significance cutoff, which is chosen at an appropriate level (e.g. based on community guidelines) for the data type and process of interest. For datasets that contain more experimental noise, additional data filtering can be performed. For example, for the viral infection-induced immune response dataset [16], we further filter out genes with <5 measurement timepoints (out of 14 total) with a significant differential expression.

*Identifying time of final expression level.* For time-series omics datasets of plasticity, we expect conservative experimental procedures where more data is collected over a (slightly) longer timeframe than the completion process itself. Moreover, for each molecular characteristic $g$ measured in the omics time series dataset, we consider the possibility that $g$ reaches its final expression level before the entire process is complete. Therefore, for each $g$, we determine the time at which final expression is reached, by evaluating each measurement ${y}_g$ against the range of ${y}_{g, final}\pm \varphi \cdot \left({y}_{g,\mathit{\max}}-{y}_{g,\mathit{\min}}\right)$, with an arbitrarily small $\varphi \in \left(0,1\right)$. In reverse temporal order, we find a set of timepoints where all consecutive ${y}_g$ is within the range as given above. Then, within this set, the *j^th^* timepoint (*j* being an arbitrary small integer, default 2) is taken as the time of final expression, ${t}_{final}$.

### Model fitting

*Calculation of*  $\tau$*.*  $\tau$ is a scaled ‘progress’ parameter that ranges from 0 to 1. For each $g$ with some measured values (or bootstrapped values, see below) over time, as a set of points in the vector space of time and the molecular measurement ${p}_{g,i}=\left({t}_i,{y}_{g,i}\right)$, first we take the scaled distance between each consecutive datapoint, $d\left({p}_{g,i},{p}_{g,i+1}\right)\cdot \lambda$. The scaling factor $\lambda$ is introduced to control the different scales between $t$ (e.g. tens to hundreds of days or hours) and $y$ (as a log2 fold change, typically within ±10). We then calculate the total distance $D={\sum}_id$, followed by calculating $\tau ={\sum}_0^i{d}_i/D$, such that $\tau =0$ at ${t}_0$, and $\tau =1$ at ${t}_{final}$.

*Data bootstrapping.* Weighted bootstrapping is chosen to address gaps in uneven time-series data, as it out-performs alternative methods such as spline-based interpolation and Gaussian process regression. The GitHub repo of this paper includes a script, compareSamplingMethods.m, to provide a visual demonstration of the comparison between these methods. Weighted bootstrapping is performed for each $g$ to generate evenly spaced datapoints prior to model fitting. We bootstrap data to be evenly spaced on $\tau$, i.e. consecutive (bootstrapped) data points have equal distances. For each bootstrapped data point, a default 1000 random samples is taken from all ${y}_g$, with measurements taken at a closer time to the bootstrap timepoint receiving higher weights. The average of the 1000 (weighted) random samples is then taken as the bootstrapped data point. By default, 100 data points are bootstrapped.

*Model fitting.* We use multiple linear regression with the binomial distribution (Equation 1) as the basis (also known as the Bernstein basis, see ref [38, 39]) to fit CP models with increasing $n$ to the *bootstrapped* data for each $g$. Of note, we take the measurements of $g$ at ${t}_0$ and ${t}_{final}$ as fixed CPs, thus for $n=1$ the model is not fitted but directly evaluated by the linear correlation between the data and the model. If this linear correlation is poor (by an arbitrary cutoff), then models for $n>1$ are fitted by multiple linear regression in the Bernstein basis, using the built-in MATLAB function `regress', which outputs $\left(n+1\right)$ number of CPs for the model ${P}_k,k=0,\dots, n$ (see Equation 2). To avoid overfitting, the maximum $n$ allowed is the number of timepoints with *measured* data for $g$. In fact, we recommend to further reduce maximum $n$ by a factor of 2 or 3; in other words, on average there should be at least 2 to 3 experimentally measured datapoints of $g$, to inform one (fitted) attractor state for $g$. In addition to preventing overfitting, this practice also reduces computational time as model fitting is the most computationally intensive step in the workflow. For each fitted model, goodness-of-fit parameters are then evaluated by the *measured* values of $g$, including the residual sum of squares (RSS); RSS penalized by a factor of ${n}^2$; and the coefficient of determination (R^2^).

*Model selection.* Based on the goodness-of-fit parameters from model fitting, the model that best fits the data can be selected by either minimum ${n}^2$-penalized RSS, or an arbitrary cutoff of RSS or R^2^. We find an arbitrary cutoff of R^2^ to be the most intuitive selection criteria, and facilitates the comparison between all $g$ within a dataset or even between datasets.

### Analysis and predictions

*Global analysis and visualization.* Following model fitting and selection, for each measured molecule $g$ we have a set of optimal CPs to describe the changes in $g$ over time, $\left\{{P}_g\right\}$. For all measured molecules in the omics dataset, then, we have $\left\{{P}_G\right\},G\ni g$. We define attractor states $S$ by the relative density of $\left\{{P}_G\right\}$ along the time-axis, where subsets of ${P}_G$ cluster more densely together on the time-axis. The clustering of ${P}_G$ on the time-axis infers both the global number of attractors as well as their timing during the completion process, while the ‘position’ of ${P}_G$ along the multi-dimensional ‘axes’ of omics data represents the molecular markers (subset of all measured molecules $G$) of each attractor $S$. This is visualized by a kernel density plot on the time-axis. Here, the timing and molecular markers of each model-identified attractor $S$ is then compared to the known markers of each phase of the plasticity programs, showing excellent agreement between the CP model and prior biological knowledge about these programs. In an unknown or understudied plasticity program, the CP model-identified attractor states would represent new knowledge discovery, which can in turn be subject to further analysis such as DAVID [34, 35] gene ontology analyses and experimental validation.

*Model predictions.* To predict the molecular outcome of interrupting the completion process at step $k$ (i.e. interrupting the transition from ${S}_{k-1}$ to ${S}_k$), each molecular characteristic $g$ is evaluated by the subset of its CPs ${P}_{g,i}$, where ${t}_i<{t}_k$. By Equation 1, this gives the dynamics of the interrupted process as predicted values in the vector space $\left(t,{y}_g\right)$, i.e. the predicted expression values of $g$ over time $t$, for $\tau \in \left[0,1\right]$. To make exact predictions of the expression value of $g$ at a precise moment $t$, we first evaluate Equation 1 in the $t$-axis to solve for the corresponding $\tau$, then calculate the expression value of $g$ by evaluating Equation 1 in the $y$-axis. For the CYHX-dataset [24], the coefficient of determination R^2^ is calculated to compare the predicted gene expression values by the experimentally measured values [24]. As comparison, a null model is constructed by a random permutation of the experimentally measured gene expression values in the CYHX-dataset [24]. R^2^ values between the null model predictions and the experimental values are calculated to be negative, indicating that the predictive power of the null model is very poor (RSS > total sum of squares). For ease of interpretation, we take these negative R^2^ values as R^2^ = 0. For the normal and EEC syndrome keratinocyte differentiation datasets [33, 37], principle components analysis (PCA) is used to compare the model-predicted gene expression profiles of three hypothetical scenarios of disease, to EEC syndrome expression data.

*Computational resources.* All procedures were implemented in MATLAB 2021a on a personal computer with a 12th Gen Intel(R) Core(TM) i7-12700H, 2.70 GHz, and 16GB RAM. Without drawing graphics, the processing time for the workflow is within minutes.

**Key Points**

- A new mathematical model and bioinformatics protocol to analyze omics time-series data.
- Model accurately identifies attractor states by their timing and molecular markers.
- Model allows non-trivial, quantitative, and time-resolved predictions, such as the molecular outcomes of blocking a plasticity program from reaching completion.

## Data Availability

The CP model is available as a suite of MATLAB functions at https://github.com/Radboud-YuLab/CPmodel. All mined data used in this paper, and scripts for data visualization, are provided at https://github.com/Radboud-YuLab/CPmodel. All procedures are implemented in MATLAB R2021a.
